# Supplementary material for: Layer-specific proteomic analysis of human hearts in patients with sudden cardiac death
Source: PLoS One. 2025 Dec 30;20(12):e0339886. doi: 10.1371/journal.pone.0339886 (PMC12753045; doi:10.1371/journal.pone.0339886)
Supplement: S1 Table — (DOCX) [file pone.0339886.s001.docx]

Supplementary Table 1. Oligonucleotide sequences for PKP2

| **Amplification** | **5’ → 3’** |
| --- | --- |
| Exon1 | ACAAGTCTCCAGGTGTCCGCG |
|  | ATAGGAGGAGGTGACCGGGTG |
| Exon2,3 | CTTGTTCTTGGCCTTCATTAC |
|  | GAACAGAATGTGCTGGCAATG |
| Exon4 | GTATTCGCTGAGTCGTCTCTG |
|  | GTTTCAGTGTGCAAAGTCACC |
| Exon5 | CATTGAATGCCTTGATGCCAC |
|  | GAGATGATGTAAGGCATCTGG |
| Exon6,7 | TCTGCCACATTTGCTGTGTTC |
|  | TCCTGACTTCCTTGGGGCTAC |
| Exon8,9,10 | AAGACCTGTTGGATACACAGC |
|  | TCCCTCACTGTTTCATCTCTG |
| Exon11 | CATCAACCTCTGGTAATCTAC |
|  | GAGGTGATACAGACAACATTTC |
| Exon12 | AACAGAGCAAGATTCCGTCTC |
|  | ATTTACACACAGGCTGGTGAG |
| Exon13,14 | GGTCTTACAGAACACCCACAG |
|  | GTTTCTTGGGCTGGGTAGTAG |
|  |  |
| **Sequencing** |  |
| Exon1 | ACAAGTCTCCAGGTGTCCGCG |
| Exon2 | CTTGTTCTTGGCCTTCATTAC |
| Exon3 | CCAGAGTAATTAGTCCTCAGC |
|  | ATGGGCAACCTCTTGGAGAAG |
| Exon4 | GTATTCGCTGAGTCGTCTCTG |
| Exon5 | CATTGAATGCCTTGATGCCAC |
| Exon6 | TCTGCCACATTTGCTGTGTTC |
| Exon7 | TCCTGACTTCCTTGGGGCTAC |
| Exon8 | AAGACCTGTTGGATACACAGC |
| Exon9 | GTCCTGTGATATCACACCTGC |
| Exon10 | TCCCTCACTGTTTCATCTCTG |
| Exon11 | CATCAACCTCTGGTAATCTAC |
| Exon12 | AACAGAGCAAGATTCCGTCTC |
| Exon13,14 | GGTCTTACAGAACACCCACAG |
