# Supplementary material for: Layer-specific proteomic analysis of human hearts in patients with sudden cardiac death
Source: PLoS One. 2025 Dec 30;20(12):e0339886. doi: 10.1371/journal.pone.0339886 (PMC12753045; doi:10.1371/journal.pone.0339886)
Supplement: Attachment — Submitted filename: Review Comments to the Author.docx [file pone.0339886.s010.docx]

Review Comments to the Author

This study, based on sudden cardiac death (SCD) cases and matched controls, presents meaningful findings. However, the overall presentation and interpretation of the data are insufficient in several key aspects.

**Merits:**
The research team enrolled SCD cases and controls over a nearly 10-year period and compared proteomic features across the three ventricular layers-an approach that adds spatial resolution and biological insight to the analysis.

**Shortcomings and Recommendations:**

1. **Layer-Specific Comparison Within Groups**
   I strongly recommend that the authors perform within-group comparisons of proteomic profiles across the three ventricular layers, particularly in the SCD group. This would help clarify whether layer-specific proteomic alterations are associated with the pathophysiology of SCD.
2. **Validation of Layer-Specific Protein Expression**
   It would be informative to simultaneously demonstrate the expression of validated proteins (e.g., PKP2, RyR2) across all three layers within individual samples. This would allow for a direct assessment of inter-layer expression heterogeneity and strengthen the spatial proteomic
3. **Protein Degradation analysis**

Postmortem autolysis can lead to significant protein degradation, potentially confounding proteomic findings. The authors should carefully evaluate and, if possible, quantify the extent of postmortem protein degradation to ensure that observed differences are primarily disease-related rather than degradation-related.

4. **Data Presentation and Statistical Clarity**

**The result section:** each subtitle should represent the main finding of the relative part. For example, line 238, this title meaningless and need to be modified according to the relative contents or findings.

**Figure 1:** The BNP levels appear markedly different between SCD and control groups. Please clarify why this difference was not statistically significant.

**Figure 2:** Provide detailed methodology for myocyte diameter measurement. How was measurement consistency ensured across different regions? Also, report the total number of myocytes analyzed per group.

**Figure 3B:** The fold changes do not appear to correspond directly to the relative abundance values. Please verify whether fold changes should be expressed as inverse values (e.g., 1/9.2 ≈ 0.11). Additionally, comparisons between SCD and CH (control heart?) groups should be included.

**Figure 4A-C:** Similar to Figure 3B, include direct comparisons between SCD and CH groups to enhance interpretability.

**Figure 5B:** The immunostaining images lack clarity in distinguishing target signals (e.g., PKP2, RyR2) from background. Consider adjusting color contrast or providing higher-resolution images. Please also specify the sample size for each group and include a comprehensive statistical summary table.

**SuppData:** I suggested to give a full list of differential expressed proteins (DEPs) among different groups and among different layers. Each data table should have a specific name.

5. **Discussion-Forensic Application Potential**
   The discussion should be expanded to address the potential utility of the identified proteins in forensic practice. A comparative analysis with existing cardiac biomarkers used in postmortem SCD diagnosis would strengthen the translational relevance of the findings.
